# Adsorption and Sensing Performance of Pt(1-3)-Modified TiSe_2_ for Dissolved Gas (CH_4_, C_2_H_2_, and CO) in Transformer Oil: A DFT Study

**DOI:** 10.3390/ijms26093985

**Published:** 2025-04-23

**Authors:** Junsheng Ding, Yingang Gui, Hua Huang

**Affiliations:** College of Engineering and Technology, Southwest University, Chongqing 400715, China; dingjunshengswu@outlook.com (J.D.); guiyingang@outlook.com (Y.G.)

**Keywords:** DFT, dissolved gas detection, Pt modification, TiSe_2_, adsorption and sensing

## Abstract

Based on density functional calculations, the adsorption and gas sensing properties of transition metal Pt(1-3)-modified TiSe_2_ for dissolved gas (CH_4_, C_2_H_2_, CO) in transformer oil were studied in this paper. Firstly, the stable structures, density of states, and energy bands of Pt(1-3)-modified TiSe_2_ were calculated. Then, the structure parameters, density of states, electrostatic potential distribution, and desorption time of Pt(1-3)-modified TiSe_2_ after adsorbing CH_4_, C_2_H_2_, and CO gas were calculated. The results show that the large binding energy between the transition metal Pt(1-3) modification and the TiSe_2_ substrate indicates that the modification systems have good structural stability. On the one hand, Pt(1-3) modification improves the conductivity of TiSe_2_. On the other hand, the transition metal Pt(1-3), which acts as the active site for gas adsorption, obviously enhances the gas adsorption effect, resulting in the significant charge transfer and a change in material conductivity. In summary, Pt(1-3)-modified TiSe_2_ significantly improves the adsorption and gas sensing performance of gas sensing materials for CH_4_, C_2_H_2_, and CO, which provides a new idea for the study of gas sensing materials for online monitoring of transformer working conditions.

## 1. Introduction

An electric power transformer plays a key role in maintaining the stability of the power system, which boosts the voltage to reduce energy loss during power transmission, and maintains voltage stability and active power balance in power distribution. However, the insulation oil and paper inside the transformer inevitably decompose to produce gas at high temperatures during long-term operation, which seriously affects the insulation [1,2,3]. It may even cause paralysis of the power system. Insulating paper is easily oxidized to produce CO at 120–130 °C during long-time working [4,5,6]. CH_4_ in insulating oil is mainly produced by partial discharge at 200 °C to 300 °C [5,7]; the internal arc discharge above 700 °C mainly produces C_2_H_2_ [5,8]. Commonly, the fault can be diagnosed by detecting the generated gas. At present, dissolved gas analysis (DGA) is mainly used for this type of transformer fault [9,10,11]. However, the DGA diagnosis method for transformers with different voltage levels has some problems, such as different standards, high detection cost, and low sensor sensitivity [12]. In this paper, gas sensing materials are constructed based on first-principles calculation [13,14], and the adsorption performance and gas sensing performance of the gas sensor for dissolved gas in transformer oil were analyzed, which provides ideas for the design of new gas sensors, and ensures the security and stable operation of the transformer.

Two-dimensional materials are commonly used as gas sensing materials [15,16]; they have the advantages of high specific surface area, sensitive electronic characteristics to environmental changes, strong surface activity, and atomic thickness [17,18]. In addition, transition metal modification on two-dimensional materials can further enhance the adsorption and gas sensing performance for gas molecules. Tang found that graphene shows higher adsorption energy, lower desorption temperatures, and better sensitivity for gas molecules after iron and manganese modifications [19]. Liu found that Ir-modified MoS_2_ was highly sensitive to the decomposition products of SF_6_, especially H_2_S [20]. A large number of studies have proved that doping or modifying transition metal atoms on the basis of two-dimensional materials can significantly enhance the adsorption effect. Pt atoms are commonly used in the field of gas sensing due to their good physical and chemical stability [21,22,23,24]. Compared with graphene and MoS_2_, the conductivity and electronic properties of single-layer TiSe_2_ are affected by the phase transition of charge density wave, and the energy gap can be completely closed after metal doping [25]. Xiao reported Cu-modified TiSe_2_ shows a good adsorption effect on CO [26]. Moreover, TiSe_2_ is a semimetal material with a smaller band gap than a common semiconductor [27,28,29].

However, few existing studies reported the application of Pt-modified TiSe_2_ in dissolved gas detection. Based on density functional theory (DFT), this study proposed one to three Pt atoms modified TiSe_2_ materials (abbreviated as Pt(1-3)-modified TiSe_2_) for the typical dissolved gases sensing: CH_4_, C_2_H_2_, and CO. By analyzing the band gaps, density of states, electrostatic potential, adsorption energy, adsorption distance, charge transfer, and desorption time, the adsorption effect and sensing performance were analyzed.

## 2. Results and Discussion

### 2.1. The Structure of the System and the Optimal Metal Modification Sites

**Figure 1:**
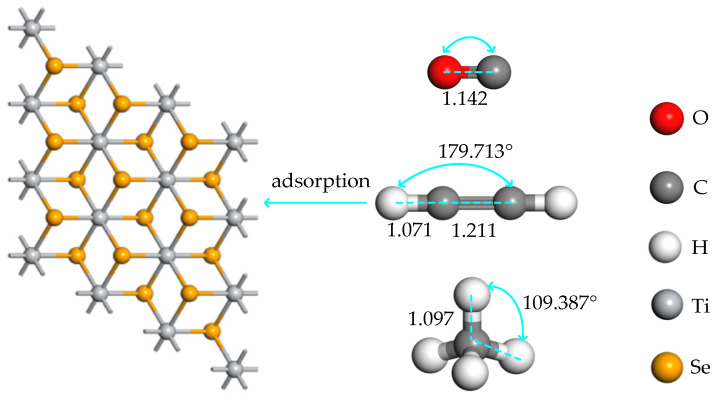
The molecular structure of the TiSe_2_ substrate and adsorbed gas, and the unit of adsorption distance is Å.

The optimized structures of the intrinsic TiSe_2_ substrate and dissolved gas molecules (CH_4_, C_2_H_2_, and CO) are shown in Figure 1. CH_4_ is a tetrahedral structure centered on the C atom, in which the C-H bond length is 1.097 Å, and the C-H-C bond angle is 109.387°. The H-C-C bond angle of C_2_H_2_ is 179.713°, and both of C_2_H_2_ and CO have a linear molecular structure with three bonds as the skeleton, the bond length of the C-C triple bond is 1.211 Å, which is greater than that of the C-O triple bond 1.142 Å, indicating that the carbon atoms of the latter are subjected to greater interatomic forces. The C-H bond of C_2_H_2_ is 1.071 Å, which is slightly smaller than that of CH_4_, indicating that the binding effect of C_2_H_2_ on H atom is greater than that of CH_4_ after structural optimization; the Ti-Se bond length is 2.57 Å, which is close to the bond length of 2.9 Å measured in previous studies [30], which verified the reliability of the calculation results.

**Figure 2:**
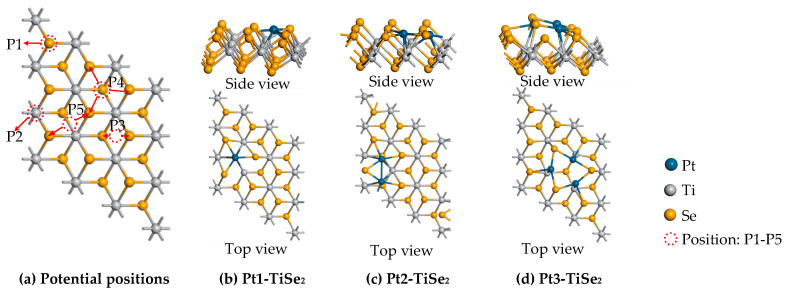
(**a**) Potential modification points and (**b**–**d**) the most stable structure of Pt(1-3)-modified TiSe_2_.

The results show that the top and hollow sites of metal atoms on TiSe_2_ have better mechanical and electronic properties [31,32]. As shown in Figure 2a, there are five potential modification positions of Pt(1-3) on TiSe_2_: top of the Ti atoms (P1 position), top of the Se atoms (P2 position), between two Ti atoms (P3 position), above the equilateral triangle formed by the top three Ti atoms (P4 position), between two adjacent Ti atoms in the same top layer (P5 position). Among these modification positions, the system with the largest metal modification binding energy was used as a gas-sensitive material for gas adsorption. The results show that the maximum E_b_ of single Pt modification (Pt1-TiSe_2_) is −4.4059 eV by P4. The maximum E_b_ of double Pt modification (Pt2-TiSe_2_) is −6.4344 eV by two adjacent P2 positions. The maximum E_b_ of triple Pt modification (Pt3-TiSe_2_) is −7.2988 eV by two Pt at P1 and P2 positions, and another Pt connected to the two Pt. The most stable modification positions after geometric optimization are shown in Figure 2b–d.

**Figure 3:**
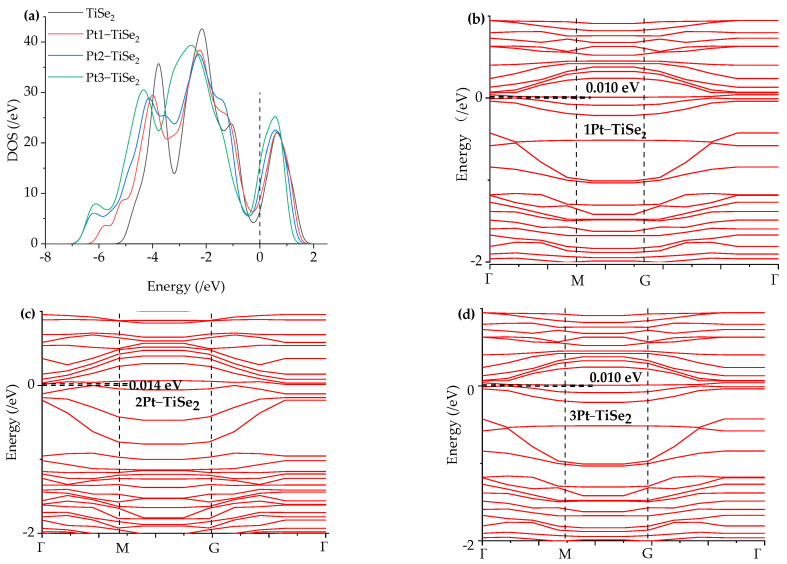
(**a**) density of states of Pt(1-3)-modified TiSe_2_ and TiSe_2_, (**b**–**d**) energy band of Pt(1-3)-modified TiSe_2_.

The density of states of intrinsic TiSe_2_ and the most stable Pt(1-3)-modified TiSe_2_ were analyzed to further explore the effect of Pt modification on the electronic properties of the TiSe_2_ system. As shown in Figure 3a, the black curve represents the density of states of the intrinsic TiSe_2_ before modification, and the red, blue, and green curves represent the density of states after modification of 1 to 3 Pt atoms, respectively. The results show that higher curves around the Fermi level 0 eV indicate enhanced electron transitions and increased conductivity. In addition, the curve moves to the left toward the lower energy, indicating that stability improves after the modification. The degree of leftward motion of the density of states near the Fermi level is Pt3-TiSe_2_ > Pt2-TiSe_2_ > Pt1-TiSe_2_, which is consistent with the magnitude of binding energy. The energy bands of Pt(1-3)-modified TiSe_2_ were shown in Figure 3b–d, the modified energy gap is only about 0.01 eV, and the electrons can easily transfer to the conduction band. The energy band near and above 0 eV is more dense, providing more positions for the electron transition. As a result, Pt atom modification increases the electron transition probability and material conductivity.

### 2.2. Gas Adsorption Property Analysis

#### 2.2.1. CH_4_ Gas Adsorption

**Figure 4:**
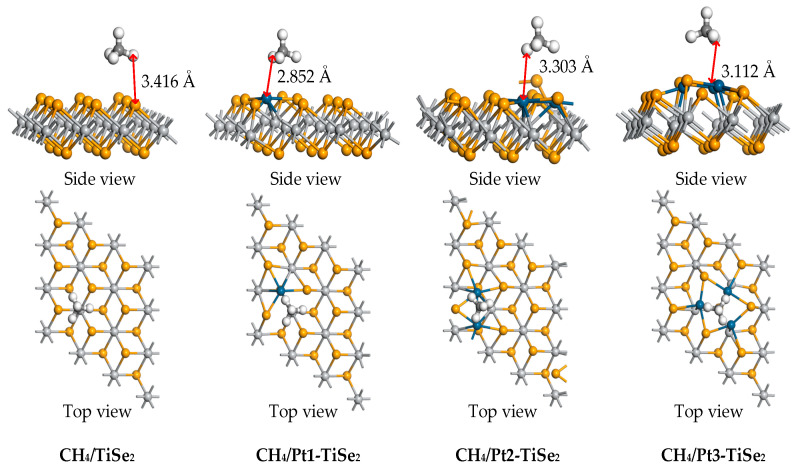
The stable structure of intrinsic TiSe_2_ and Pt(1-3)-modified TiSe_2_ for adsorbing CH_4_ gas.

The CH_4_ adsorption calculation was performed based on the most stable Pt(1-3)-modified TiSe_2_. Due to the different spatial relative positions of gas molecules and Pt(1-3)-modified TiSe_2_, the adsorption energy, charge transfer, and adsorption distance of the most stable Pt(1-3)-modified TiSe_2_ are shown in Table 1. The most stable adsorption structures of intrinsic TiSe_2_ and Pt(1-3)-modified TiSe_2_ for CH_4_ are shown in Figure 4. Table 1 shows that the maximum adsorption energy of CH_4_/TiSe_2_ system is −0.297 eV, and the maximum adsorption energy of CH_4_/Pt(1-3)-modified TiSe_2_ systems are −0.250 eV, −0.248 eV, and −0.270 eV, respectively, indicating there are few changes in the adsorption energy of CH_4_ before and after modification. However, the adsorption distance between CH_4_/TiSe_2_ and CH_4_/Pt(1-3)-modified TiSe_2_ system decreased from 3.416 Å to 2.852 Å, 3.303 Å, and 3.112 Å, respectively. The interaction between the gas molecules and the substrate was stronger after modification, which can also be confirmed by the charge transfer. The CH_4_ charge transfer before and after modification is −0.054 e, −0.062 e, −0.060 e, and −0.061 e, respectively. CH_4_ receives electrons during adsorption and obtains more electrons through the transfer with the modified Pt atoms. Besides, the charge transfer of the metal before and after adsorption is also analyzed; the electrons lost by Pt are 0.028 e, 0.043 e, and 0.052 e. According to the charge transfer of CH_4_ and Pt atoms, the electron transfer is most active upon 3 Pt modification, which also corresponds to the large adsorption energy (−0.270 eV) and small adsorption distance (3.112 Å).

**Figure 5:**
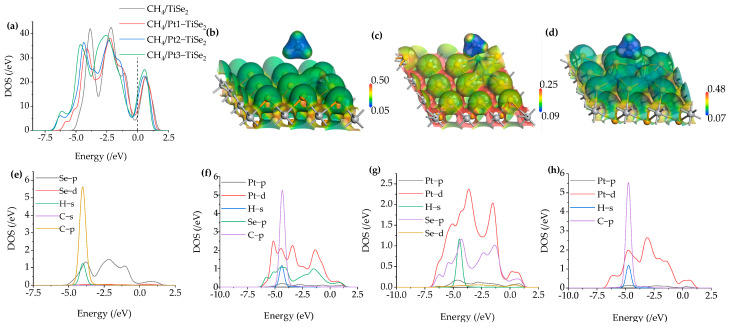
(**a**) The TDOS of CH_4_/TiSe_2_ and CH_4_/Pt(1-3)-modified TiSe_2_, (**b**–**d**) The ESP of CH_4_/Pt(1-3)-modified TiSe_2_, (**e**–**h**) The PDOS of CH_4_/TiSe_2_ and CH_4_/Pt(1-3)-modified TiSe_2_.

The total density of states (TDOS), partial density of states (PDOS), and electrostatic potential (ESP) of the adsorption system are analyzed to further analyze the molecular interactions during the adsorption process, as shown in Figure 5. In Figure 5a, the red, blue, and green curves represent TDOS after modifying 1 to 3 Pt. Compared with the black curve of intrinsic TiSe_2_, the curve moves up after the 1 to 3 Pt modification near the Fermi level, resulting in an increase in conductivity, which is also consistent with the charge transfer in Table 1. The TDOS of the modified system showed new peaks between −7.5 eV and −5 eV, and the combined PDOS analysis showed that these peaks are due to the modified Pt-d orbital. For PDOS of CH_4_/TiSe_2_ in Figure 5e, the interaction of Se-p, H-s, and C-p orbits is strong between −5 eV and −2.5 eV. The PDOS curves of CH_4_/Pt1-TiSe_2_ in Figure 5f indicate that there is orbital hybridization between Pt-p, H-s, and C-p. The PDOS of CH_4_/Pt2-TiSe_2_ in Figure 5g shows an interaction of H-s with Se-p. The PDOS of CH_4_/Pt3-TiSe_2_ in Figure 5h shows that Pt-d hybridizes with C-p. Figure 5b–d shows the ESP of the CH_4_/Pt(1-3)-modified TiSe_2_ system, and blue to red represents from low to high potential. The results show that the potential around C in CH_4_ is lower than the substrate potential, and the potential on the H atom surface is similar to that of the substrate. Also, the substrate and gas potential do not overlap, indicating that the charge transfer during adsorption tends to be between H atoms and the substrate.

#### 2.2.2. C_2_H_2_ Gas Adsorption

**Figure 6:**
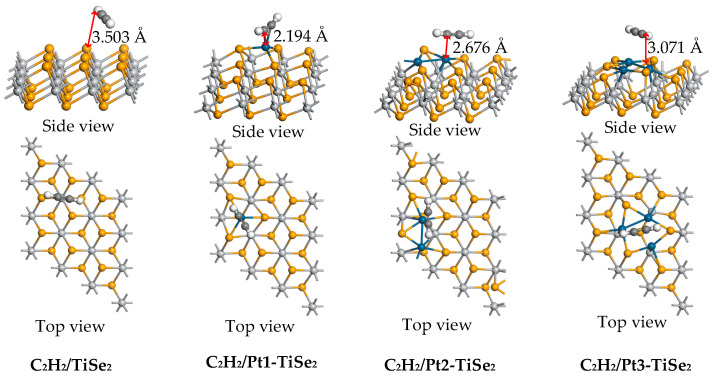
The stable structure of intrinsic TiSe_2_ and Pt(1-3)-modified TiSe_2_ for adsorbing C_2_H_2_ gas.

The parameters and structures of the most stable CH_4_/TiSe_2_ and CH_4_/Pt(1-3)-modified TiSe_2_ systems are shown in Table 2 and Figure 6. The maximum adsorption energy of C_2_H_2_/TiSe_2_ for C_2_H_2_ gas is −0.405 eV, and the maximum adsorption energy of C_2_H_2_/Pt(1-3)-modified TiSe_2_ is −0.940 eV, −0.489 eV, and −0.422 eV, respectively. Among them, the adsorption energy of C_2_H_2_ was most significantly improved after one Pt modification. The adsorption distance between the substrate and the gas molecules before and after modifying 1 to 3 Pt decreased from 3.503 Å to 2.194 Å, 2.676 Å, and 3.071 Å, which enhanced the intermolecular force and favored the gas adsorption. The amount of C_2_H_2_ charge transfer in C_2_H_2_/TiSe_2_ and C_2_H_2_/Pt(1-3)-modified TiSe_2_ systems is −0.019 e, 0.031 e, 0.053 e, and −0.004 e, respectively. In addition, the electrons lost by Pt were 0.080 e, 0.032 e, and 0.031 e, respectively. Among them, the metal atoms transfer the most electrons after modifying one Pt. Considering the C_2_H_2_ charge transfer and metal charge transfer, Pt1-TiSe_2_ has the most active electron transfer during C_2_H_2_ adsorption, which also corresponds to the shortest adsorption distance of 2.194 Å and the maximum adsorption energy of 0.940 eV. This shows that TiSe_2_ modified with one Pt can comprehensively improve the adsorption behavior of C_2_H_2_.

**Figure 7:**
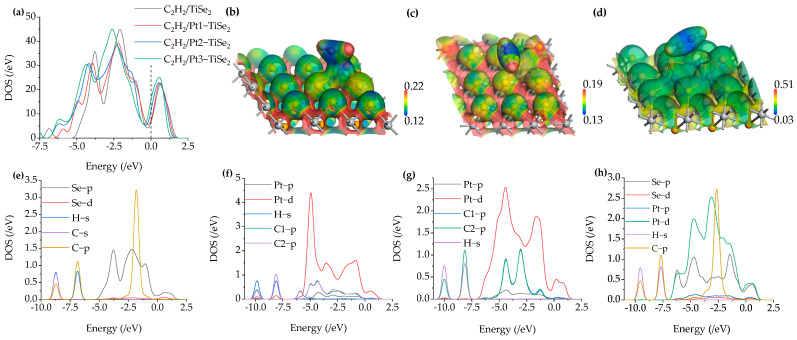
(**a**) The TDOS of C_2_H_2_/TiSe_2_ and C_2_H_2_/Pt(1-3)-modified TiSe_2_, (**b**–**d**) The ESP of C_2_H_2_/Pt(1-3)-modified TiSe_2_, (**e**–**h**) The PDOS of C_2_H_2_/TiSe_2_ and C_2_H_2_/Pt(1-3)-modified TiSe_2_.

The TDOS and PDOS after C_2_H_2_ adsorption are shown in Figure 7a and Figure 7e–h, respectively. Figure 7a shows that the TDOS curve of C_2_H_2_/Pt(1-3)-modified TiSe_2_ system shifts up at the Fermi level compared with C_2_H_2_/TiSe_2_, and the conductivity of the system enhances after Pt modification. Figure 7e–h show that the peaks of the TDOS curve near −5 eV, −2.5 eV are mainly from the contribution of the C-p orbit and the Pt-d orbit. However, the peaks of TDOS in C_2_H_2_/Pt1-TiSe_2_ and C_2_H_2_/Pt2-TiSe_2_ systems decrease at these two energy levels due to the hybridization of Pt-p orbital and C1-p, C2-p orbitals. Figure 7e shows the hybridization of C-p and H-s orbitals near −7.5 eV, and the interaction of Se-p, Se-d, and C-p orbitals appears near 0 eV. In addition, a new peak appeared between −7.5 eV and −5 eV after Pt modification, according to the corresponding energy level in PDOS, Figure 7f shows that the reason is the hybridization between the p orbitals of the two C atoms and Pt-d orbital after adsorption, Figure 7g shows that reason is the highest Pt-d orbital, and Figure 7h shows that the reason is the hybridization between the Se-p and Pt-d orbitals.

The ESP of the C_2_H_2_/Pt(1-3)-modified TiSe_2_ system is shown in Figure 7b–d, respectively, where the color of blue to red indicates the potential from low to high. The results show that the potential on the H surface is higher than that around C. The potential distribution around the two C atoms in the C_2_H_2_ molecules of the C_2_H_2_/Pt1-TiSe_2_ and C_2_H_2_/Pt2-TiSe_2_ systems is not identical, but the potential of the C atoms is basically the same in the C_2_H_2_/Pt3-TiSe_2_ system. The red color on the H atom surface is lighter than the former two, indicating that the potential is lower. Additionally, Figure 7b shows that the potential distribution of C_2_H_2_ after adsorption overlaps with that of a modified Pt, which indicates that the gas and substrate are more active in the adsorption process, and the electron transfer is easier. In addition, the blue color above the overlap is darker than the blue color below, indicating a lower upper potential, and the electron moves from a lower potential to a higher potential. Thus, the gas loses electrons, which is consistent with the loss of 0.031 e at the maximum adsorption energy position after one Pt modification in Table 2.

#### 2.2.3. CO Gas Adsorption

**Figure 8:**
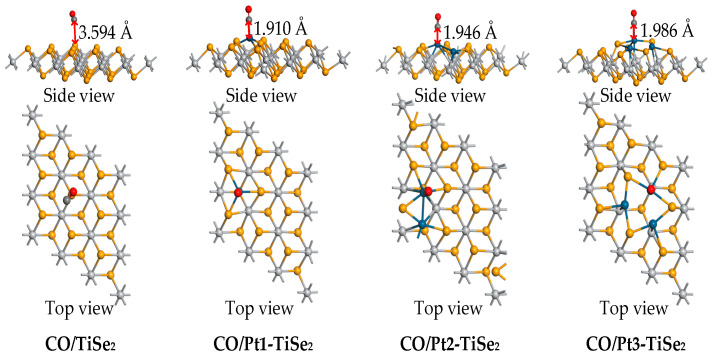
The stable structure of intrinsic TiSe_2_ and Pt(1-3)-modified TiSe_2_ for adsorbing CO gas.

The adsorption parameters and adsorption structures of intrinsic TiSe_2_ and Pt(1-3)-modified TiSe_2_ for CO gas at the maximum adsorption energy are shown in Table 3 and Figure 8. The maximum adsorption energy of the CO/TiSe_2_ system and the CO/Pt(1-3)-modified TiSe_2_ system changed from −0.219 eV to −1.338 eV, −0.851 eV, and −0.703 eV, respectively, which significantly improved the adsorption energy, especially after one Pt was modified. Correspondingly, the adsorption distance decreased from 3.594 Å to 1.910 Å, 1.946 Å, and 1.986 Å, respectively. The amount of CO charge transfer before and after modification was −0.002 e, 0.001 e, 0.001 e, and 0.004 e, which shows that the amount of CO charge transfer was very small. In addition, the metal atoms lost electrons during the adsorption process, and the charge transfer amounts were 0.034 e, 0.003 e, and 0.001 e, respectively, indicating that one Pt modification lost many more electrons. Therefore, by combining the charge transfer of CO and Pt, it is found that the charge transfer is the most active after modifying one Pt, which is consistent with the shortest adsorption distance of 1.910 Å and the maximum adsorption energy of −1.338 eV.

**Figure 9:**
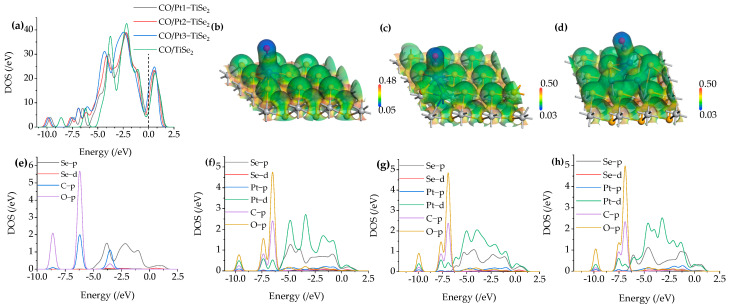
(**a**) The TDOS of CO/TiSe2 and CO/Pt(1-3)-modified TiSe_2_, (**b**–**d**) The ESP of CO/Pt(1-3)-modified TiSe_2_, (**e**–**h**) The PDOS of CO/TiSe_2_ and CO/Pt(1-3)-modified TiSe_2_.

TDOS and PDOS after CO adsorption are shown in Figure 9a and Figure 9e–h, respectively. The green curve in Figure 9a corresponds to the TDOS curve of the intrinsic TiSe_2_ after CO adsorption, while the black, red, and blue curves correspond to the TDOS curve of CO adsorption after 1 to 3 Pt-modified TiSe_2_, respectively. Figure 9a shows that the black, red, and blue curves near the Fermi level 0 eV are slightly higher than the green curves, indicating that the conductivity enhances during the adsorption process after modifying Pt. According to the PDOS curve in Figure 9e–h, it is found that the Pt-d and Se-p orbits coincide at 0 eV, and the Pt-p, Se-d, C-p, and O-P orbits also coincide at 0 eV, indicating that electrons are shared to enhance conductivity. Figure 9e shows that in the CO/TiSe_2_ system, C-p is hybridized with Se-p orbits between −5 eV and −2.5 eV. Furthermore, peaks were observed in the TDOS curve near −2.5 eV and −4 eV, which were mainly contributed by Se-p and Pt-d orbitals, according to the PDOS. However, the two peaks of the TDOS curve of CO/Pt(1-3)-modified TiSe_2_ are decreased at these two energy levels. Figure 9f–h show that the decrease may possibly be caused by the hybridization between the Se-d and the O-p orbit near −2.5 eV, and the hybridization between Pt-p and O-p near −4 eV. It is also found that the TDOS curves of CO/Pt(1-3)-modified TiSe_2_ showed new peaks at −7.5 eV and 10 eV compared with that of CO/TiSe_2_, and the PDOS curve found that it was mainly caused by Pt-d, C-p, and O-p orbitals.

The ESP of the most stable CO/Pt(1-3)-modified TiSe_2_ systems is shown in Figure 9b–d. Compared with the adsorption of CH_4_ and C_2_H_2_, the potential distribution of the gas and substrate upon CO adsorption has overlap after 1-3 pt modifications, which indicates that there is a more active role of electrons between CO and substrate in the adsorption process, which is consistent with the shorter adsorption distance of CO than CH_4_ and C_2_H_2_. Similarly, the overlap of C and Pt(1-3)-modified TiSe2 is observed; the upper site is yellow, and the bottom is green, indicating that the upper site has a higher potential. Thus, the metal loses electrons due to the movement direction of electrons from low to high potential. By comparison, the color span of the overlapping parts in Figure 9b–d is smaller than Figure 7b, indicating smaller potential difference and weaker electron movement, so compared to the C_2_H_2_/Pt1-modified TiSe_2_ shown in Figure 7b where the metal loses 0.080 e, the metal in CO/Pt(1-3)-modified TiSe_2_ loses fewer electrons. Moreover, the blue color on the surface of O in the CO molecule is deeper, indicating that its potential is lower than that of the C atom and the substrate. During the adsorption process, the electrons of O move from the gas molecule to Pt(1-3)-modified TiSe_2_, which is consistent with the loss of electrons when the adsorption energy of CO is maximum after 1-3 Pt modifications in Table 3.

### 2.3. Gas Desorption Property Analysis

**Figure 10:**
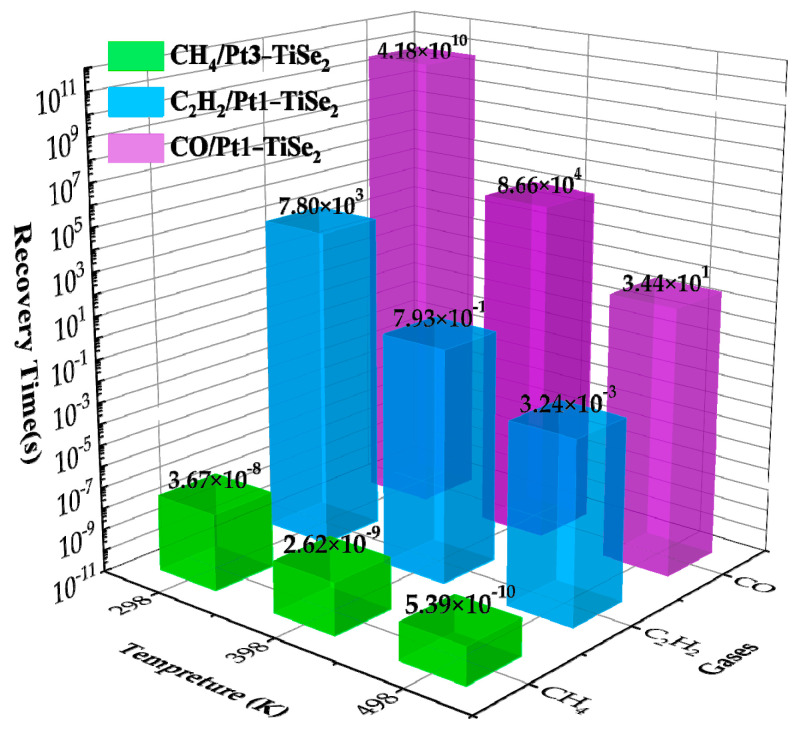
The desorption time of CH_4_, C_2_H_2_, and CO/Pt(1-3)-modified TiSe_2_ system with maximum adsorption energy at temperatures of 298 K, 398 K, and 498 K.

Pt(1-3)-modified TiSe_2_-based materials should not only show moderate adsorption capacity to dissolved gas molecules, but the gas should exhibit good desorption ability to ensure the reusability of Pt(1-3)-modified TiSe_2_-based materials. For CH_4_, C_2_H_2_ and CO, the desorption time was calculated by using the adsorption energy of the most stable adsorption structure of Pt(1-3)-modified TiSe_2_ for dissolved gas in oil at 298 K, 398 K, and 498 K, to evaluate the reusability of gas sensitive materials, as shown in Figure 10. From Equation (3), the higher the temperature, the shorter the desorption time; the greater the absolute value of adsorption energy, the longer the desorption time. Therefore, the desorption time increases for a large adsorption energy system at room temperature. An equilibrious desorption time can be reached when the temperature rises, then a good gas sensing response/recovery can be achieved in this temperature range. In Figure 10, the adsorption energy of CH_4_ is very small, but the desorption time at the minimum temperature is still minimal, so the system is not the best choice to detect CH_4_. For C_2_H_2_ and CO, it has a large adsorption energy, but the desorption time decreases to 0.8 s and 34 s when the temperature increases to 398 K and 498 K, respectively. Therefore, the Pt(1-3)-modified TiSe_2_-based gas-sensitive material has good response/recovery performance for C_2_H_2_ and CO gas.

## 3. Calculation Methods

All calculations are performed based on the density functional theory of the DMol^3^. TiSe_2_ adopts a triangular prismatic phase crystal of P3m1 space group with a structural shape of Se-Ti-Se triple layers [33,34]. The TiSe_2_ supercell was built by 3 × 3 × 1 primitive cells along the (0 0 1) direction, and the vacuum layer was set to 20 Å to prevent adjacent TiSe_2_ layers from interacting in the z direction. Structure optimization is used to obtain the most stable structure of the system, and the function perdew-burke-ernzerhof (PBE) and generalized gradient approximation (GGA) were selected [35,36]. During the geometry optimization process, the convergence standard of self-consistent field is 1 × 10^−5^ Ha, the maximum atomic force between atoms is 0.002 Ha/Å, and the maximum atomic displacement is 0.005 Å. The number of sampling points of the Brillouin area is set to 7 × 7 × 1 [37,38].

For Pt(1-3)-modified TiSe_2_, the metal atoms show different modification positions for each modification mode. By calculating the stable structure of different modification positions, the modification position with the maximum binding energy is used for gas adsorption. The binding energy *E_b_* is calculated by Equation (1). *E_Metal-TiSe_*_2_ represents the total energy of the system after the Pt(1-3) metal modification, *E_TiSe_*_2_ represents the energy of the intrinsic TiSe_2_, and *E_Metal_* represents the energy of the metal atoms. Due to the different adsorption positions between dissolved gas molecules and Pt(1-3)-modified TiSe_2_, the adsorption energy (*E_ads_*), adsorption distance, and charge transfer (*Q_t_*) are calculated to select the most stable adsorption structures. The adsorption energy *E_ads_* is calculated by Equation (2). *E_gas/metal-TiSe_*_2_ represents the total energy after gas adsorption on Pt(1-3)-modified TiSe_2_, *E_gas_* represents the total energy of the gas molecule, and *E_Metal-TiSe_*_2-*max*_ represents the total energy of Pt(1-3)-modified TiSe_2_. The recovery time is calculated by Equation (3), which represents the time required for the gas response to recover from 100% to 10%. *ω* represents the trial frequency, which represents the number of times per second that molecules attempt to desorb from the adsorption sites on the surface, typically valued at 10^12^ s^−1^ for solid surfaces. *T* is Kelvin temperature, and *K_B_* is Boltzmann constant (8.62 × 10^−5^ eV/K) [39,40,41,42].

(1) $$E_{b}=E_{Metal-TiSe_{2}}-E_{TiSe_{2}}-E_{Metal},$$

(2) $$E_{ads}=E_{gas/Metal-TiSe_{2}}-E_{gas}-E_{Metal-TiSe_{2}-\max},$$

(3) $$\tau =\omega^{-1}\cdot \exp\left(-E_{ads}/TK_{B}\right)$$

## 4. Conclusions

Based on DFT calculations, the behavior of intrinsic TiSe_2_ and Pt(1-3)-modified TiSe_2_ adsorption to CO, CH_4_, and C_2_H_2_ was investigated. Firstly, the structures of intrinsic TiSe_2_, Pt(1-3)-modified TiSe_2_, dissolved gases, and gas adsorption were constructed. Then, the gas adsorption and sensing properties of gases on intrinsic TiSe_2_ and Pt(1-3)-modified TiSe_2_ are studied by analyzing the adsorption TDOS, PDOS, ESP, and desorption time. In summary, Pt(1-3) atoms form stable binding structures on the TiSe_2_ surface with binding energy in the order of Pt3-TiSe_2_ > Pt2-TiSe_2_ > Pt1-TiSe_2_. The energy band gaps of the Pt(1-3)-modified TiSe_2_ system are about zero, and the energy band of the conduction band around the Fermi level becomes denser. The DOS of Pt(1-3)-modified TiSe_2_ systems increases and moves left near the Fermi level. As a result, Pt(1-3) atom modification increases the conductivity of TiSe_2_. Pt(1-3) atom modification increases the adsorption capability of TiSe_2_ to CO, CH_4_, and C_2_H_2_. Particularly, Pt1-TiSe_2_ has the maximum adsorption energy for CO (−1.338 eV) and C_2_H_2_ (−0.940 eV). However, Pt(1-3)-modified TiSe_2_ shows weak adsorption capacity to CH_4_. Combining with the TDOS, PDOS, and ESP analysis, the conductivity of the adsorption systems significantly changes during the adsorption of CO, CH_4_, and C_2_H_2_, resulting in good gas sensing properties. In Figure 5a, Figure 7a, and Figure 9a, the TDOS curves after adsorption of the three gas molecules show a significant increase near the Fermi level, indicating a decrease in the sensor’s resistance response. This provides theoretical support for the sensor’s sufficient sensitivity. The desorption time analysis shows that the desorption time of CH_4_ was very small at all temperatures; the desorption time of C_2_H_2_ is 0.8 s at 398 K, and the desorption time of CO is 34s at 498 K. Overall, Pt1-TiSe_2_ has good gas response/recovery performance for dissolved gas in transformer oil. The results of this study lay the foundation for the experimental preparation of a gas sensor for online monitoring and fault diagnosis of transformer failure.

## Figures and Tables

**Table 1:** Adsorption parameters of the intrinsic TiSe_2_ and Pt(1-3)-modified TiSe_2_ for CH_4_ gas.

System	E_ads_ (eV)	*Q_t_* (e)	Distance (Å)
CH_4_/TiSe_2_	−0.297	−0.054	3.416
CH_4_/Pt1-TiSe_2_	−0.250	−0.062	2.852
CH_4_/Pt2-TiSe_2_	−0.248	−0.060	3.303
CH_4_/Pt3-TiSe_2_	−0.270	−0.061	3.112

**Table 2:** Adsorption parameters of the intrinsic TiSe_2_ and Pt(1-3)-modified TiSe_2_ for C_2_H_2_ gas.

System	E_ads_ (eV)	*Q_t_* (e)	Distance (Å)
C_2_H_2_/TiSe_2_	−0.405	−0.019	3.503
C_2_H_2_/Pt1-TiSe_2_	−0.940	0.031	2.194
C_2_H_2_/Pt2-TiSe_2_	−0.489	0.053	2.676
C_2_H_2_/Pt3-TiSe_2_	−0.422	−0.004	3.071

**Table 3:** Adsorption parameters of the intrinsic TiSe_2_ and Pt(1-3)-modified TiSe_2_ for CO gas.

System	E_ads_ (eV)	*Q_t_* (e)	Distance (Å)
CO/TiSe_2_	−0.219	−0.002	3.594
CO/Pt1-TiSe_2_	−1.338	0.001	1.910
CO/Pt2-TiSe_2_	−0.851	0.001	1.946
CO/Pt3-TiSe_2_	−0.703	0.004	1.986

## Data Availability

Data are contained within the article.
